# The bee, the flower, and the electric field: electric ecology and aerial electroreception

**DOI:** 10.1007/s00359-017-1176-6

**Published:** 2017-06-24

**Authors:** Dominic Clarke, Erica Morley, Daniel Robert

**Affiliations:** School of Biological Sciences, University of Bristol, Life Science Building, 24, Tyndall Avenue, Bristol, BS8 1TQ UK

**Keywords:** Bees, Plants, Electric fields, Pollen, Mechanoreception

## Abstract

Bees and flowering plants have a long-standing and remarkable co-evolutionary history. Flowers and bees evolved traits that enable pollination, a process that is as important to plants as it is for pollinating insects. From the sensory ecological viewpoint, bee–flower interactions rely on senses such as vision, olfaction, humidity sensing, and touch. Recently, another sensory modality has been unveiled; the detection of the weak electrostatic field that arises between a flower and a bee. Here, we present our latest understanding of how these electric interactions arise and how they contribute to pollination and electroreception. Finite-element modelling and experimental evidence offer new insights into how these interactions are organised and how they can be further studied. Focussing on pollen transfer, we deconstruct some of the salient features of the three ingredients that enable electrostatic interactions, namely the atmospheric electric field, the capacity of bees to accumulate positive charge, and the propensity of plants to be relatively negatively charged. This article also aims at highlighting areas in need of further investigation, where more research is required to better understand the mechanisms of electrostatic interactions and aerial electroreception.

## Introduction

**Fig. 1:**
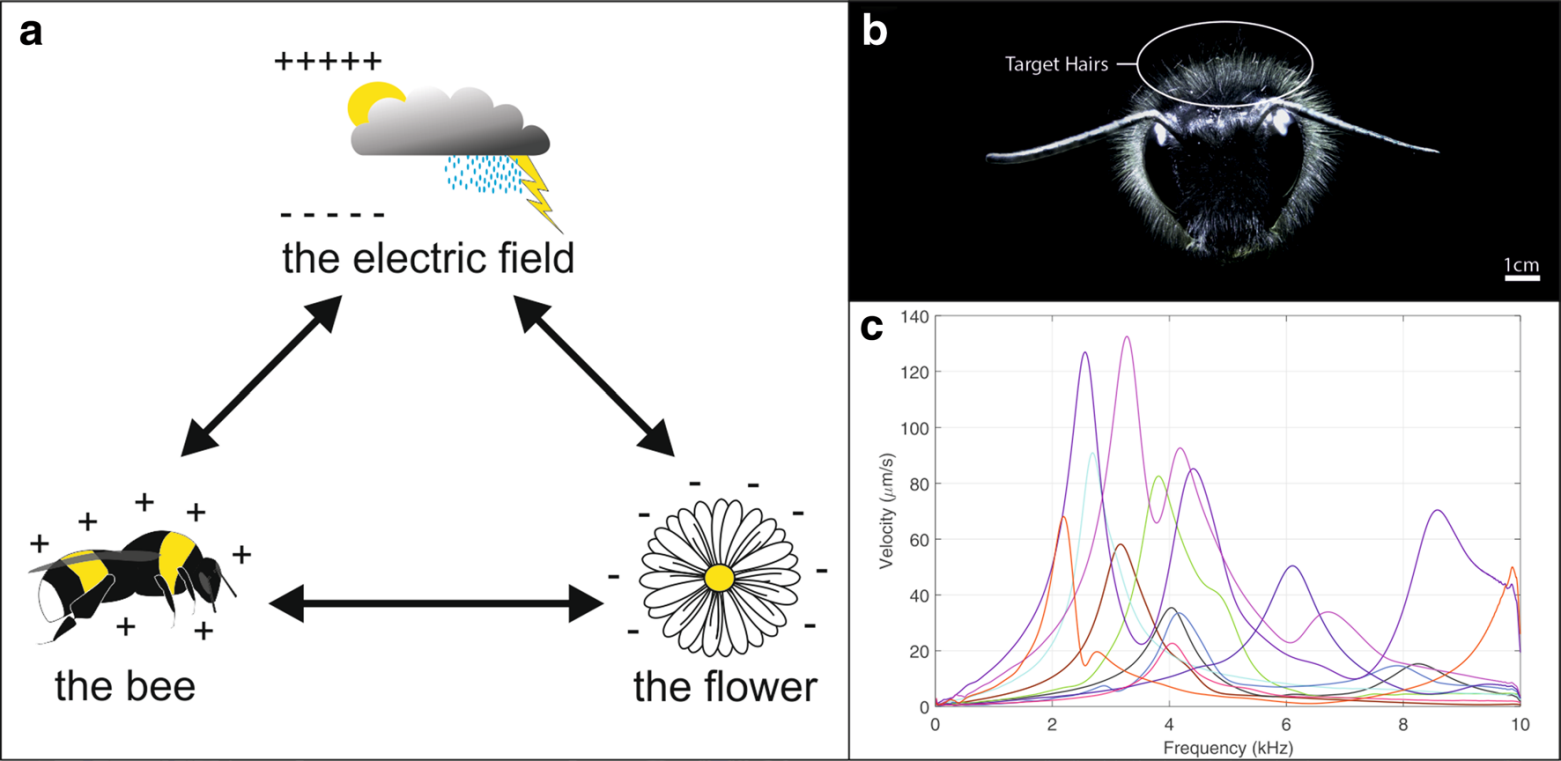
Electromechanical reception in bumblebees and electrical ecology of pollination. **a** Interactions between bee, flower, and atmospheric electric field cannot be separated, as each of them influence the other. **b** Frontal confocal photograph of a bumble bee illustrating target hairs used to establish the site of electromechanical detection of electric fields in bumble bees (modified from Sutton et al. 2016). **c** Mechanical response of diverse bumble bee hairs to an electric field stimulus. Vibration velocity *v* elicited by a multifrequency electrical stimulus (0–10 kHz) was measured using laser Doppler vibrometry. Each *line* represents a measurement of a single hair from different animals (*N* = 12). Hair displacement *d* as a function of frequency *f* is given by *d* = *v*/*πf*

Animal pollinators detect and select flowers by their colours, shapes, patterns, fragrant volatiles (Raguso 2008), and, in some cases, temperature (Rands and Whitney 2008) and tactile cues (Kevan and Lane 1985). Some plants can signal to their pollinators using corolla air humidity (von Arx et al. 2012) and even using acoustics, as some bats use floral echoes to localize nectar resources (Simon et al. 2011). The diversity of animal pollinators, and the variety of pollination syndromes, is vast (for a comprehensive review, see Willmer 2011). While bees carry out fewer total flower visits than other pollinators, they are responsible for about half of all crop pollination (Rader et al. 2016). The relationship between bees and flowers constitutes a complex example of co-evolutionary adaptation, whereby the interests of both parties are served. Flowers use bees as vehicles to enhance pollen transport and fertilization, while bees greatly benefit from pollen and nectar as food sources (for a recent review, see Nicholls and Hempel de Ibarra 2017). This co-evolutionary relationship turns out to be rich and complex. It involves cooperation between plants and their animal vectors, to the benefit of each, but also involves competition and adaptive compromise. For example, the nectar reward is expensive for the plant to produce. In milkweed, up to 37% of the daily photosynthetic energy is spent producing nectar reward for pollinators (Southwick 1984), energy that is no longer available to the plant. However, a more energetic reward is likely to attract more pollinators, forcing a trade-off. Literature on the subject is very rich and diverse, exploring the complexities of plant-pollinator interactions, and the diverse strategies and adaptations for plant reproduction and insect foraging (Chittka and Thomson 2001). Here, we present an aspect of plant–pollinator interactions that has been underappreciated thus far: the presence of electrostatic forces between bees and flowers. We describe how and why these electric fields exist, the mechanism by which bees detect weak aerial electrostatic forces and the function of these forces in pollen transfer (Fig. 1a).

## Detection of weak electric fields in air by bees

Electroreception, defined as the ability of an organism to detect external electric forces, has long been known in animals living in aquatic, electrically conductive environments, for example, in fish (Kalmijn 1971; Bullock and Heiligenberg 1986), in amphibians (Hurd et al. 1984), and in platypus (Gregory et al. 1987). Although electroreception in an aerial environment had previously been hypothesised in bees (Yes’kov and Sapozhnikov 1976; Corbet et al. 1982), only recent studies have provided behavioural and physiological evidences for the phenomenon (Clarke et al. 2013; Greggers et al. 2013; Sutton et al. 2016). So far, evidence points to detection mechanisms for aerial electric forces that are very different from those described in aquatic animals (Bullock and Heiligenberg 1986). In aquatic animals, electroreception relies on direct transmission of stimulus from the conductive medium (water) to the nervous system, via conductive receptor channels (ampullae of Lorenzini). For terrestrial animals residing in air, an electrically resistive medium, detection of electric fields must operate differently and constitute a new sensory capacity.

Bumble bees, *Bombus terrestris* (Clarke et al. 2013) and honey bees, *Apis mellifera* (Greggers et al. 2013) have been shown capable of detecting weak electric fields, each in different behavioural contexts, using different sensory mechanisms. Bumble bees can sense the presence of weak electric fields (e-fields) surrounding flowers, and discriminate between e-fields with different radial geometries (Clarke et al. 2013). The sensory basis for e-field detection in bumble bees appears to rely on mechanosensory hairs, which are mechanically deflected by an applied electric stimulus (Sutton et al. 2016) (Fig. 1b, c). The mechanical deflection of these hairs in turn elicits neural responses, conveying information to the bee’s central nervous system. Using laser Doppler vibrometry (a technique that measures nano-scale vibrations), the deflection of a series of dorsal hairs in response to applied electric fields reveals a collective sensitivity covering a range of stimulus frequencies (Fig. 1c).

Bumble bees can use electric information to discriminate between rewarding and unrewarding flowers (Clarke et al. 2013). They can also learn colour discrimination tasks faster when colour cues are paired with electric field cues similar in magnitude to those surrounding natural flowers. In effect, bumble bees did not learn as readily in an electrically neutral environment (Clarke et al. 2013). This evidence establishes that electric fields are part of the sensory experience of foraging bees, one of the many, multimodal floral cues. It seems, therefore, that floral e-fields are used to inform foraging behaviour in bumble bees. Honey bees reportedly use electroreception for intraspecific communication, utilising their antennae in e-field detection (Greggers et al. 2013). Unlike bumblebees, returning honey bee foragers perform a waggle dance, which communicates details of food sources to other conspecifics within the hive (von Frisch 1954). In addition to sensory cues in other modalities, low-frequency oscillating electrical stimuli are produced by electrically charged vibrating foragers as they perform the waggle dance. Honey bees are sensitive to these stimuli (Greggers et al. 2013).

The sensory basis for electroreception in honey bees was hypothesised to be the antennae, electro-mechanically coupled to the surrounding e-field in virtue of bees being electrically charged, and thus subject to electrostatic forces. Transduction of these forces was proposed to be taking place in the antennal Johnston’s organ (Greggers et al. 2013). Greggers et al. (2013) demonstrate that honey bee antennae oscillate under electric field stimulation, and this stimulation can elicit activity in the antennal nerve. It was also shown that honey bees with removed or fixed antennae are less able to associate food reward with electric field stimuli within a classical conditioning paradigm. Investigating the sensory basis for electroreception in bumblebees, it was shown that the electromechanical sensitivity (i.e., velocity and angular displacement in response to electric field stimuli) of bumble bee hairs is considerably greater than that of their antennae (Sutton et al. 2016). The sensitivity of these hairs to a wide variety of stimulation was reported in direct comparison with the antennae of the same individual bees. Hairs were shown to move with roughly an order of magnitude greater velocity and 3–4 orders of magnitude greater angular displacement than antennae across a broad frequency bandwidth. Peak response of the hairs typically occurred at stimulus frequencies between 2 and 4 kHz, consistent with the low mass and high stiffness of the hairs. The minimum electric field required to produce measurable deflections in the hairs was between 0.77 and 61 V/m depending on stimulus frequency. Antennae required larger minimum field strengths of between 15.3 and 306 V/m. Extracellular recordings from the base of the hairs in 15 individual bumble bees show increased neural activity accompanying electric field stimuli even at low frequencies (<1 Hz) (Sutton et al. 2016).

In both hairs and antennae, the mechanism of electric field detection in bees is thought to be the same: a rigid cantilever, projecting from the body, carrying an electric charge, subject to external electric force, with a sensitive force-transducer at the base. The motion of both of these structures is lever-like, where the whole structure displaces in constant phase with linearly increasing amplitude from base to tip, and minimal bending (see supplementary materials for Sutton et al. 2016). This arrangement simultaneously maximises the electric charge density at the tip of the lever (increasing electrostatic force at the tip) and the moment arm of the force with respect to the fixed base of the lever. Therefore, it seems that although bumble bees and honey bees can both detect weak e-fields, they do so using different but physically analogous electromechanical systems.

Neither mechanosensory hairs nor antennae are unique to bees. It is, therefore, possible that other arthropods also use these structures to detect electric fields. Mechanosensory structures in insects have previously been shown to be involved in detection of various fluid-mechanical signals, by a diverse range of arthropods (Casas and Dangles 2010). These signals include the air displaced by moving prey or predators, or the air flowing over the body of the animal during flight. Mosquitoes use their antennae to detect sound particle velocity in lieu of tympanal organs for audition (Göpfert et al. 1999). In the case of fluid-mechanical signals, the signal source must be viscously coupled to the sensory structure through the medium. In the case of electroreception, the signal source is directly electrically coupled to the sensory structure through the electric field. Furthermore, electric field detection is not subject to the same boundary layer constraints that influence fluid-mechanical signal detection (Casas and Dangles 2010). Both electroreception and fluid-flow detection are comparable, and indeed, electrostatic actuation has been used to generate deflections of the particle velocity and fluid-flow sensors in fruit flies and scorpions (Hoffmann 1967; Albert et al. 2007). Electrical stimuli elicit a neural response from hair deflections of 0.04° in bumble bees (Sutton et al. 2016). Minimum sensitivity thresholds have yet to be established. This is within the same order of magnitude as cricket filiform hair sensitivity, which shows neural responses to hair deflections of 0.02° (Shimozawa et al. 2003). This suggests that electroreception by mechanosensory hairs or antennae is no more demanding a sensory task than fluid-flow detection by the same structures.

Electroreception by hairs and antennae needs not to be mutually exclusive. This sensory ability may be called upon in different behavioural and ecological contexts, either by different species in different environments, or by individuals performing different roles within and outside the hive. For all currently known examples of aerial electroreception, the exact nature of the information transferred remains elusive. However, the results of the above-cited experiments (Clarke et al. 2013; Greggers et al. 2013; Sutton et al. 2016) point to the possibility that bees can detect and use aerial electric fields in the contexts of foraging and in-hive communication over short distances (none of the above studies demonstrate e-field detection at distances higher than 10 cm). Other behavioural functions are not excluded, but have yet to be investigated.

## Unveiling the nature of electric ecology

Understanding any sensory system requires not just a description of ability with an associated sensory structure, but a detailed understanding of the external stimuli in the environment of the organism. The structure, distribution, and abundance of *information* available to an organism are of comparable importance to that of energy and reproductive opportunity. For vision, a great deal is known about both the physiological underpinnings of the sense, and the world of external stimuli relevant to it (Cronin et al. 2014). This is also true of audition and olfaction (Barth and Schmid 2001). For aerial electroreception, the picture is less well developed. Relatively little is known about the structure and dynamics of electric fields and electric charges at the spatiotemporal scale of flowers and bees. This is a scale which is too large and heterogeneous to describe in precise mathematical terms and too small to measure accurately with the conventional equipment, especially in the context of field biology. Furthermore, the conventional electronic measuring equipment does not perform well when applied to extremely high impedance measurement regimes such as measuring electric fields in air. That is to say that there is no good equivalent of the camera or the microphone to acquire spatially resolved static or dynamic information about weak electric fields and their interactions with living organisms.

In the following sections, we present our current understanding of the interactions between the bee, the flower, and the aerial electric field, and spell out some emerging questions. How and under which circumstances do electric fields arise in the environment and around living matter? How and why can these fields be relevant for sensory detection and perception? What are the signals being sent and received by organisms within this modality? What are the magnitudes of the forces involved? What environmental factors affect the proper functioning of this sense? Can we effectively describe the dynamic electrical environment of plants and their pollinators?

## The electric field: atmospheric electricity and the atmospheric potential gradient

The atmospheric potential gradient (APG) is the term given to the vertical electric field that exists between the earth and the upper atmosphere (reviewed in detail in Rycroft et al. 2000, 2012). The APG is of great importance to the electrical ecology of biological systems, as will be outlined below and in the following sections. To understand its effects on plants and animals, we first provide a brief overview explaining the APG itself.

**Fig. 2:**
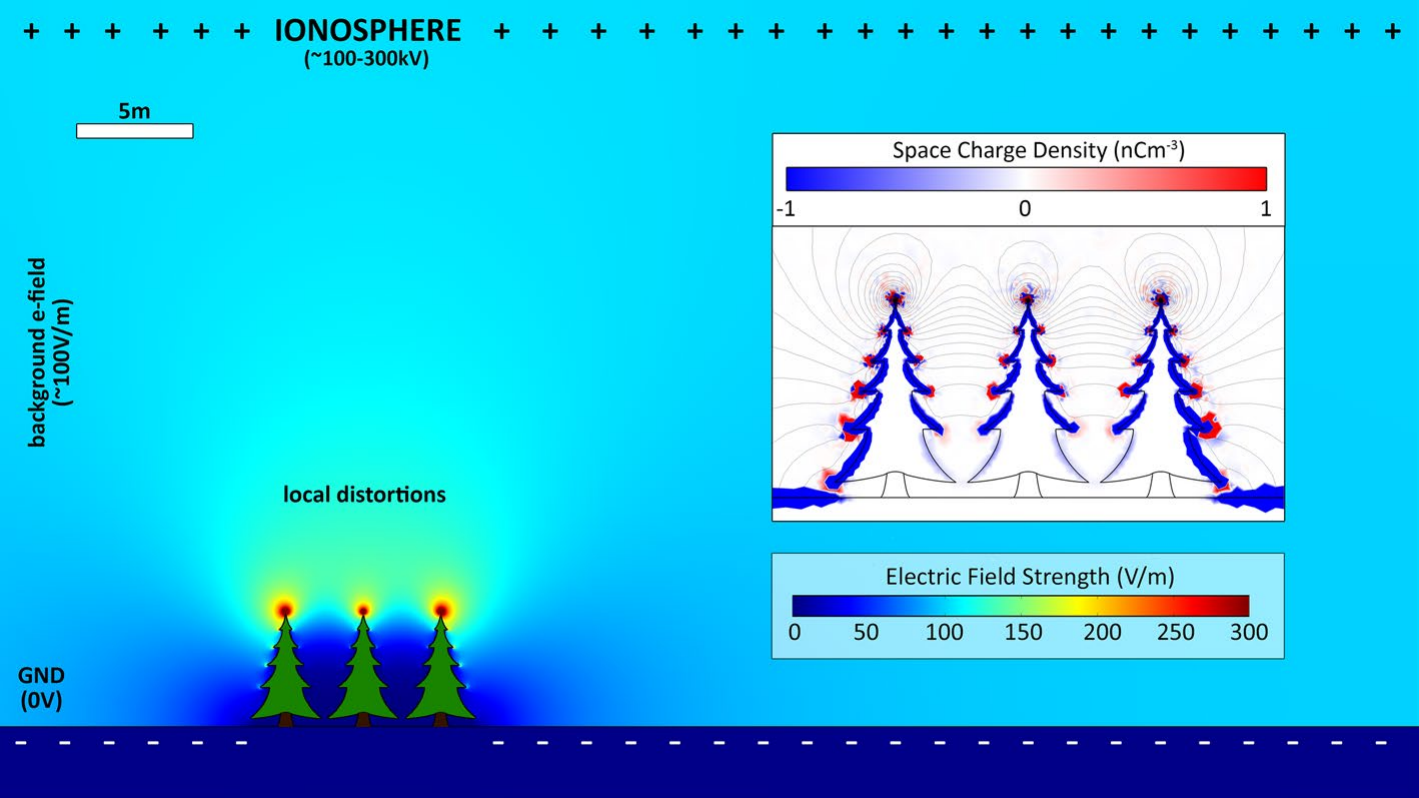
Finite-element model of the atmospheric potential gradient (APG) and its interaction with plants. *Background colour* shows strength of the electric field as a function of altitude from ground (scale *inset*). The positive ionospheric charge is included, located between 60 and 100 km above ground (not to scale). Local distortions in the uniform APG, caused by the presence of the trees, are labelled. The approximate space charge density is shown *inset*. The APG induces negative charges to build up on the surface of the ground and, in higher density, on the upper surfaces of the trees, which, in turn, induce opposite charges in the surrounding air

The electric field strength at the surface of the earth, in a flat, open region, in fair weather, is of the order of 100 V for every 1 m gained in altitude (~100 V/m). This increase in voltage continues towards a maximum of around 300 kV at an altitude of 30–50 km, above which the potential begins to decrease and invert (Beach and Rines 1904). This potential gradient is maintained globally by the action of electrical storms taking place around the earth. The current that flows down to earth in the fair weather field is exactly balanced by lightning strikes moving charge in the opposite direction elsewhere on the planet (Rycroft et al. 2000, 2012). Collectively, these processes are known as the global atmospheric electric circuit. In response to the positive potential of the air, negative charge accumulates on the surface of the earth. This charge accumulation results from electrostatic induction, whereby a charged object external to a conductor induces the opposite charge to move to that conductor’s surface, proximal to the external charge (Feynman et al. 1964). This charge accumulation cancels out the electric field inside the conductor. Likewise, negative charges inside the earth accumulate on the surface to cancel out the 100 V/m field just above the surface. The surface of the earth behaves rather like a single plate of a capacitor, where the opposite plate is the high-voltage layer of upper atmosphere and the dielectric is the air. Any object that is conductively linked to earth accumulates negative charge at its surface in this way. The further into the field this object extends, the greater the potential difference between its upper surface and the surrounding air (~100 V for every meter). This effect results in large concentrations of charge that, in turn, produce their own electrical forces, generating local distortions in the otherwise uniform atmospheric electric field (Fig. 2).

Using a mathematical model, 5 m tall trees growing on flat ground are shown to produce substantial alterations in the electric field strength in the surrounding air. The positive charge in the atmosphere draws a negative charge to the surface of the trees (Fig. 2, inset). Notably, sharp extremities and spikes produce larger fields than shallow curves or flat surfaces. This is because the charge density at the surface of a conductor is inversely proportional to the radius of curvature of that surface (Feynman et al. 1964). The higher the charge density at a point, the larger the electric field is around this point. In effect, local distortions to the APG caused by grounded objects are not only influenced by object height, and APG strength, but also very much by the geometry of the object. These effects also apply at smaller scales. At the scale of the flower, we name the local distortions in background e-field around a flower, the “floral electric field” (Clarke et al. 2013). This is the scale at which bees are acting and interacting. Understanding the dynamics of electric fields at this scale is, therefore, essential to understanding the electric sense of bees and the electrical signalling of flowers.

## The Bee: the role of the triboelectric effect

Bees are not electrically connected to the earth, like flowers are, yet they also gain a charge as they fly through the air. The acquisition and maintenance of charge on a bee is a key factor in their ability to detect electric fields (Sutton et al. 2016), with just slight increases in charge causing large gains in electromechanical sensitivity of both hairs and antennae. It is also crucial to intraspecific electrical communication in honey bees; the dancing bee must be charged to convey electrical signals to fellow bees within the hive (Greggers et al. 2013). A bee’s bulk charge will also induce stronger electrical interactions between itself and any flower it visits, strengthening electrostatic forces on pollen. The mechanism by which bees gain their charge, however, is not well understood.

The triboelectric effect is the name given to the phenomenon of materials either taking on or giving up electrons upon frictional contact with a different material, thus becoming negatively or positively charged, respectively. Each material can be placed on a series, with more positive-going materials at one end and more negative-going materials at the other. This is called the triboelectric series. While the phenomenon is somewhat variable and difficult to quantify, the relative position of a large number of synthetic and organic materials on this series is fairly well understood (Diaz and Felix-Navarro 2004). Walking insects have been shown to gain charge through friction between their bodies and the surface on which they walk (Edwards 1962). By measuring the polarity of these charges, Edwards (1962) was able to determine that insects have a propensity to gain a positive charge upon frictional contact with various materials, placing them on the positive end of the triboelectric series along with other biological materials such as human skin and cat fur.

The tendency of bees to become positively charged is well evidenced (Erickson 1975; Vaknin et al. 2000; Clarke et al. 2013). This charge generates forces on other charged objects in the vicinity of the bee from flowers to the electroreceptor hairs and antennae of conspecifics. The net charge on the bee was measured as between around +30 and +50 pC depending on the particular study, and genus. The size and shape of bees suggest a very low capacitance (~pF), such that surface voltage would be of the order of hundreds of volts (Yes’kov and Sapozhnikov 1976; Corbet et al. 1982). If the charge was concentrated in certain areas (e.g., sensory structures like hairs and antennae), this potential could be much greater. It is a reasonable hypothesis that triboelectric interactions play a role in this charging. The high-energy motion of flight is met with friction from the air and between various surfaces on the bee itself.

**Fig. 3:**
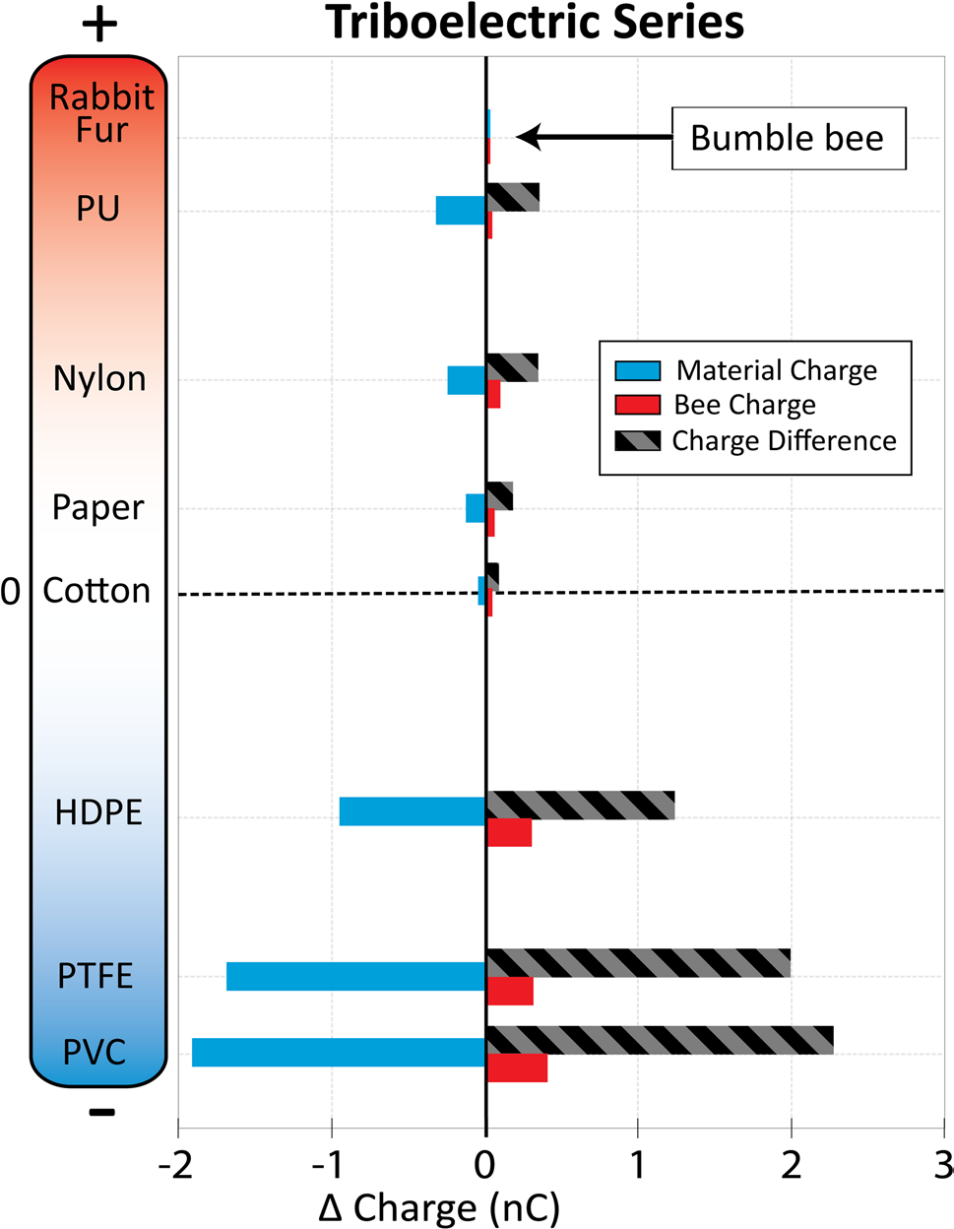
Position on the tribolelectric series of various materials, including bumble bees and rabbit fur. *Y*-axis shows the position of each material according to available literature (Diaz and Felix-Navarro 2004). Materials shown are rabbit fur, polyurethane foam (PU), nylon, paper, cotton, high-density polyethylene (HDPE), polytetrafluoroethylene (PTFE), and polyvinyl chloride (PVC). Those at the *top* (*red*, positive) end tend to lose electrons during frictional contact with other materials, becoming positively charged. Those at the *bottom* (*blue*, negative) tend to gain electrons and become negatively charged. The *x*-axis shows the difference in charge of the material (*light blue*) and a bumble bee (*red*) after rubbing the material on the dorsal surface of the bee. The *grey* and *black* striped *bars* show the bee charge minus the material charge, giving the total charge transferred triboelectrically during the rubbing. No material caused a bumble bee to become negatively charged after contact, placing the bee at the most positive end of the triboelectric series along with rabbit fur. Each datum shown is an average of ten repetitions of the measurement

To determine the triboelectric rank of bees, we have conducted a similar experiment to Edwards (1962), rubbing the dorsal surface of freshly killed bees against various materials, both synthetic and natural. We included several materials that are often found at the most positive end of the series like rabbit fur and polyurethane foam. We also used various other materials that occupy positions across the series from positive to negative. Before each measurement, bee charge was zeroed, and then, the bees were manually rubbed along a 10 cm strip of material, ten times each, at which point the charge on the bee and the material were both measured using a Faraday pail as in Clarke et al. (2013). Measurements were carried out at 50% relative humidity and 20 °C, and each measurement was repeated ten times. We were unable to identify a material that caused the bee to gain a negative charge, placing the bee at the far positive end of the series (Fig. 3). Edwards’ (1962) results on moths, beetles, and flies reveal the same tendency for insect cuticle to charge positively, where only asbestos (not available in our experiments) became more positively charged than the insects.

The results of the triboelectric experiments raise several questions: Is the tendency to become positively charged generic to all flying insects, or does it constitute an adaptation particular to bees or pollinators? Triboelectric charging is not simply a function of the material’s intrinsic electrical properties, but also of surface morphology which affects the contact surface area undergoing friction. Is the insect cuticle physically adapted to maximise or modulate this effect? Does charge separation or equalisation take place when bees experience contact and friction against flowers that have conductive connections to ground? All of these questions pose difficult experimental challenges that have not been fully addressed.

## The flower: floral electric field

**Fig. 4:**
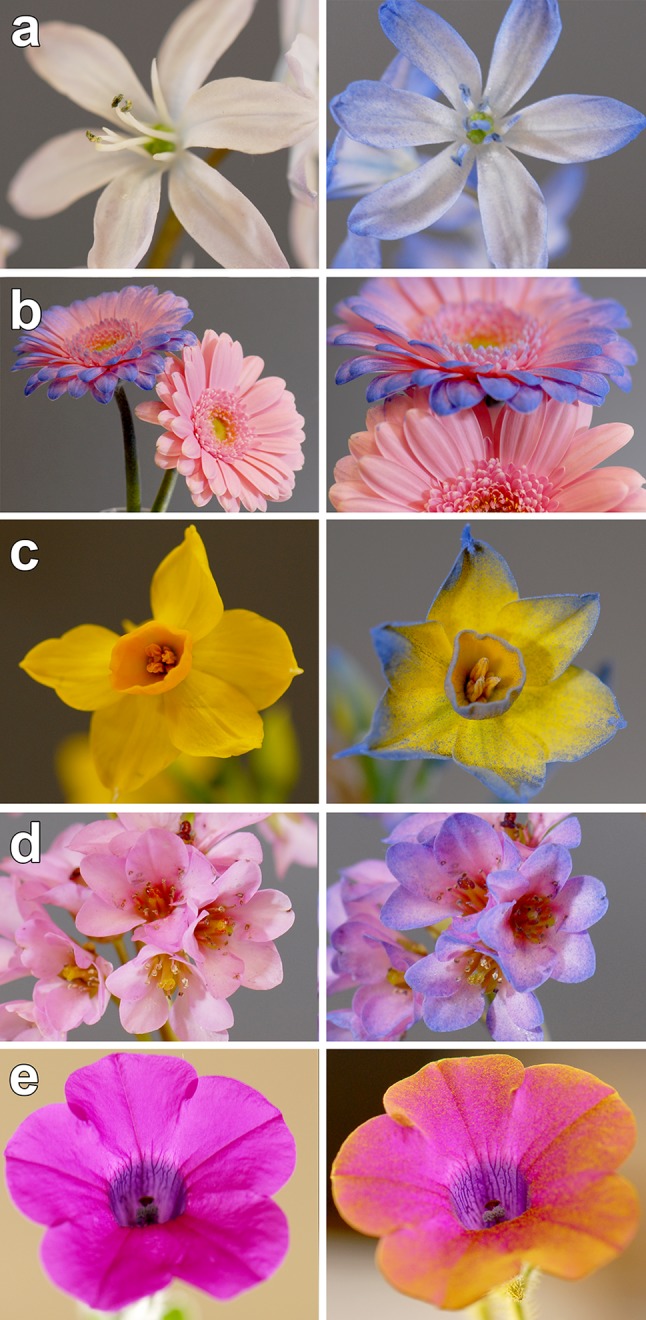
Experimental visualisation of floral electric field using electrostatic dusting. Flowers are shown before (*left*) and after (*right*) dusting with positively charged coloured powder (*blue* or *yellow* on the *bottom* image). Genera shown are **a** *Lilium*, **b** *Gerbera*, **c** *Narcissus*, **d** *Bergenia*, and **e** *Petunia*. Heterogeneous powder deposition reveals spatial differences of charging in the flower, with higher levels of deposition corresponding to points of greater negative charge density on the flower. The principal determinant of shape and strength of the floral electric field is flower geometry. Flowers were connected to electrical ground and no APG was experimentally imposed

An aesthetic if not quantitative way of visualising the presence of an electric field around a flower can be achieved by dusting natural flowers with electrostatically charged coloured powder (Clarke et al. 2013). The structure of the electric field near to the surface of a flower is revealed by the selective deposition of coloured charged dust (Fig. 4). Some details of floral anatomy are highlighted with noticeably larger paint deposition, whereby the parts of the flower with higher charge density (and therefore greater electric force) tend to be along contours and sharp features, such as the edges of petal, stigma, and anthers, but also small features such as the trichome (Fig. 4). This method of visualising heterogeneity in floral charge density highlights the possibility that flower anatomy plays a role in building a structured electric field. In turn, this opens up questions about the actual, species-specific electrical characteristics (e.g., conductivity and capacitance) of different floral organs and their potential role in insect/flower interactions. The difference in charge density between different structures can be thought of as electrical “contrast”. The geometry of petal edges, where charge density is high, provides spatial structure to the floral field, giving it an outline. There is further contrast between the flat petal surfaces and the protruding trichomes, stigma, and anthers. Just like they display spectacular corolla adaptations for certain pollination syndromes, different plant species may employ different strategies in the way they organise their electrostatic floral footprint, to make themselves electrically distinctive to potential pollinators. Spiked flowers such as teasels (*Dipsacus* sp.) should have a distinct pattern of electrical contrast from a petunia (*Petunia* sp.) for example, and likewise, a raceme will have a different electrical contrast to an umbel inflorescence. Features with sharp edges or points, both within a flower (anthers, stigma, petal edges, etc.) or on the overall plant (racemes, single protruding blooms), should have a stronger electric field purely due to their geometry.

Computer simulation can help quantify electrical forces without the need for direct measurements. In particular, finite-element analysis (FEA) allows the partial specification of a physical situation using empirical data, which can then be used to calculate many difficult or impossible to measure features of that situation. For aerial electroreception, these models stand in where direct measurement of the electric field is unavailable, providing a description of how the morphology and material properties of flowers and bees interact to induce forces on one another, and on charged objects in their vicinity. In Fig. 4, we show a model of positively charged bumble bees, flying in the vicinity of *Petunia* flowers rooted in the ground. While this model is simplified for illustration, the simulation is still empirically specified, with plant material properties, bee charge, and atmospheric electrical conditions taken from available literature. These 2D models are easily generalised to 3D (Clarke et al. 2013), with the biggest challenge being the specification of the intricate 3D geometry of flowers.

In this model (Fig. 4), the colour of the air shows the magnitude of the electric field vector at that point in space. Contour lines are lines of constant electric potential. Where they are close together, the electric field strength is high. The strongest electric fields arise as a bee approaches close to a flower. The bee carries a positive charge that it acquires while flying (Colin and Chauzy 1991; Clarke et al. 2013). The flower gains a negative charge through electrostatic induction. The forces that exist between charges fall off with the square of their distance (Feynman et al. 1964), so that the forces between bee and flower increase quickly as the bee approaches. At a distance of 2–3 cm shown in Fig. 4, the region between the bee and the flower has an electric field of over 5 kV/m. This is comparable in magnitude to the electric field at the ground under a high-voltage power line. This field strength is much higher than that required to elicit mechanical and neural responses from bumble bee hairs (Sutton et al. 2016). Hence, forces that produce detectable fluctuations in bee hairs arise at several body lengths away from the flower. These are the forces that bees can detect to assess floral rewards.

With currently available instrumentation, it is not possible to directly measure the computed quantities with as good a spatial or temporal resolution as that provided by finite-element analysis. A further benefit conferred by the finite-element analysis is that it allows experimenters to quickly explore the wide parameter space that exists within the specification of the model, changing and tweaking many aspects of it and comparing the results of thousands of simulated experiments. This can be extended from 2D into 3D, and from stationary to dynamic, time-domain, and frequency-domain studies. Ultimately, a dynamic representation of the electrical scene involving flying bees, growing plants, and an ever-changing atmospheric potential gradient could be constructed.

## Interactions on smaller scales: electrostatic pollen transfer

**Fig. 5:**
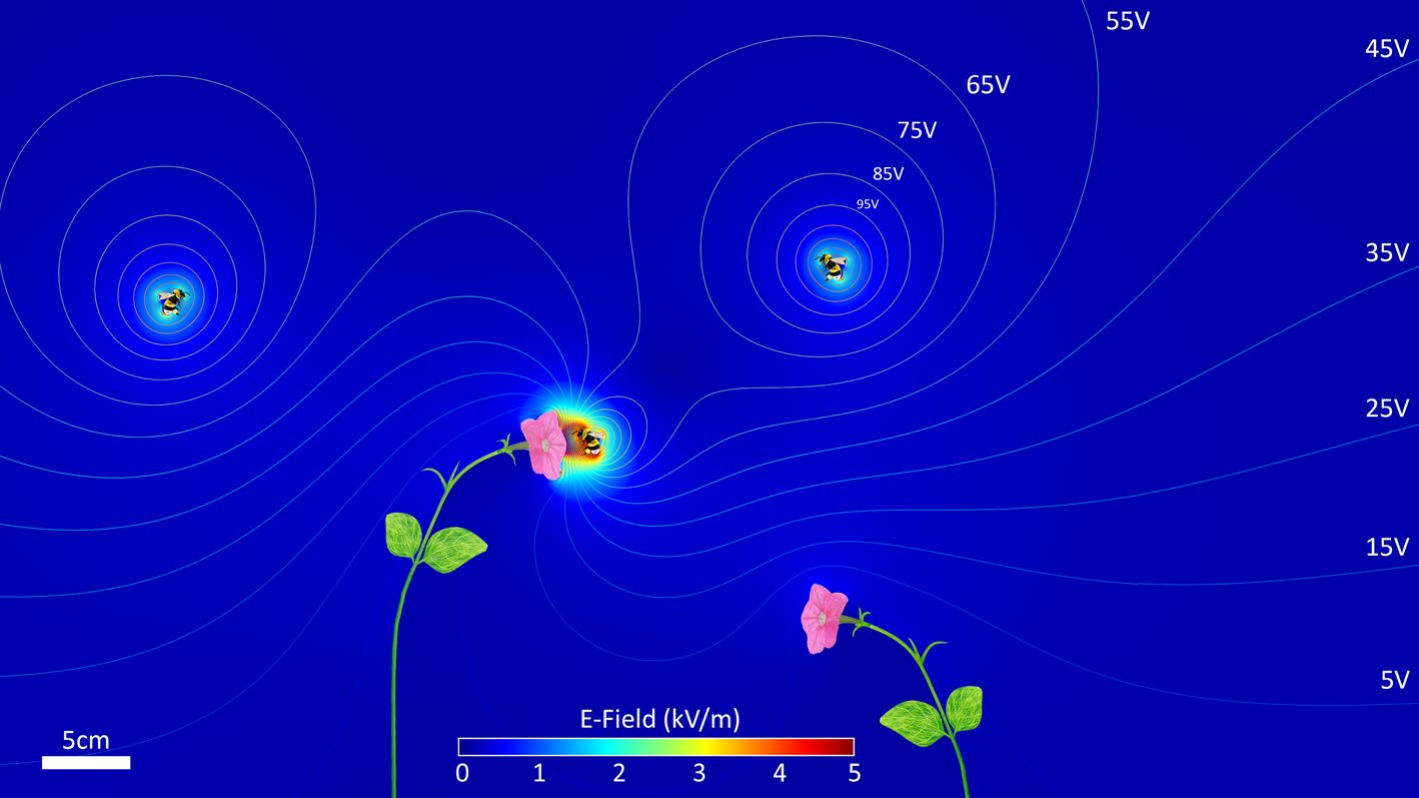
Visualising electric ecology. Finite-element model of electric interactions between positively charged bumble bees and grounded petunias (*Petunia* sp.) against the background of the atmospheric potential gradient. Bees are modelled as a simplified silhouette of their body shape—ignoring the fine structure of their appendages and hair cover. Total bee charge was taken as 32 pC, unevenly distributed on its surface area, with higher charge densities on high curvature areas, such as the head, wings, and abdomen. Petunias are modelled as slightly electrically conductive bodies that are grounded to earth (resistivity ~10 MΩm). Model petunias are constructed to mimic their natural shape. The electric field strength is encoded as the colour of the air domain (see *scale bar*, *bottom-centre*), and *contour lines* show every 10 V interval in electric potential. For a bee near a petunia (centre of image), electric field strength becomes much larger (>5 kV/m) as a positive charge is brought towards a negative charge, with a good insulator (air) preventing currents from flowing to counter-act the force. In this modelled scene, both insects and plants influence the structure and magnitude of the electric field. Electric field is zero everywhere inside the conductive regions (bees, petunias)

**Fig. 6:**
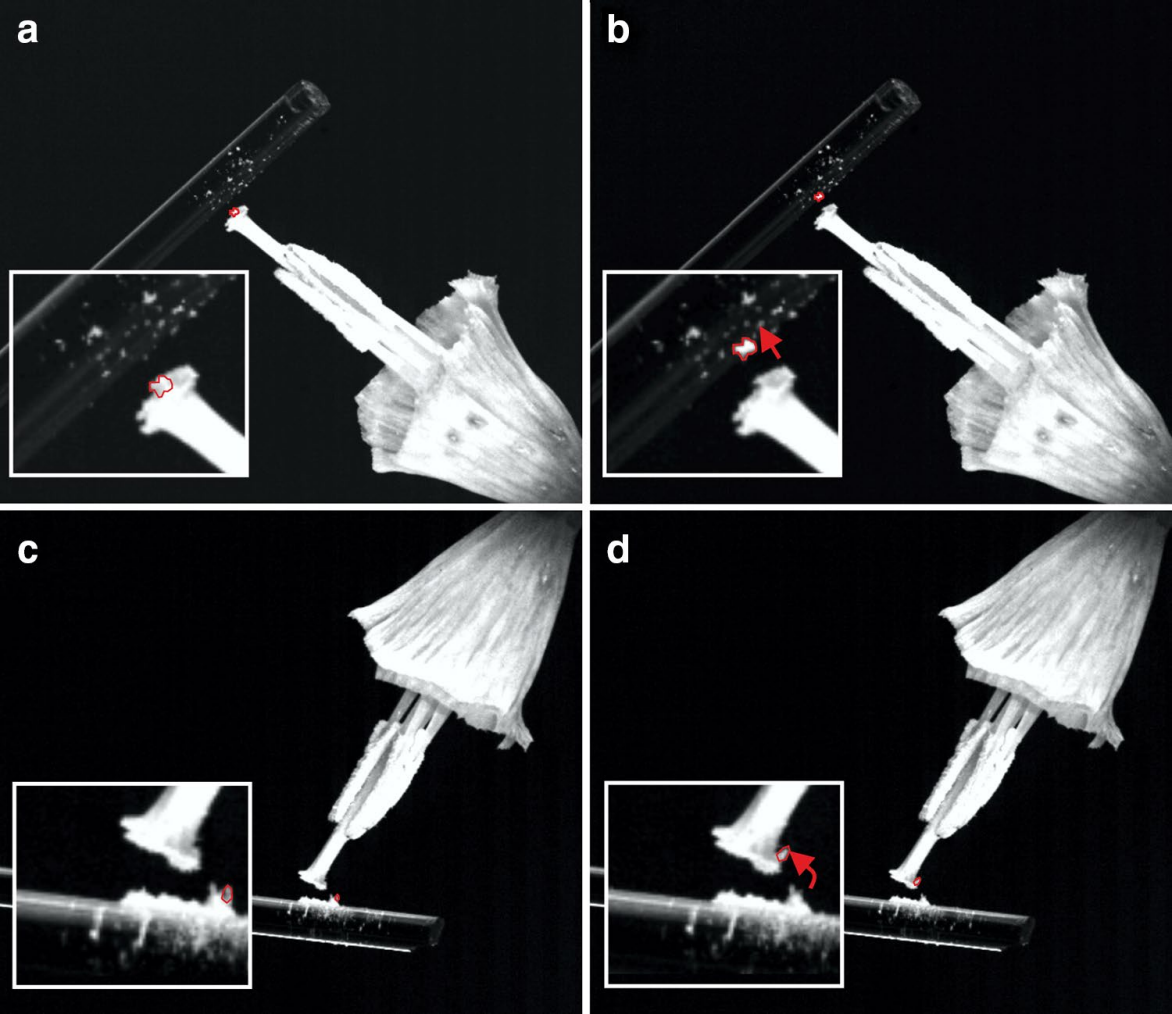
Bidirectional pollen transfer in an electric field. Daffodil (*Narcissus* sp.), with corolla removed was exposed to the electric field produced by a triboelectrically charged acrylic rod, and pollen grain motion was observed. **a** Charged rod is approached to the flower. Pollen on stigma is highlighted in *red*, with close-up of stigma (*inset*). **b** Following the approach of the charged rod, pollen jumps and attaches to the rod (*red arrow*, *inset*). **c** Electrostatically positively charged rod loaded with Daffodil pollen, with highlight of a grain (*red outline*, *inset*). **d** After approaching the stigma with a pollen laden rod, the pollen grain jumps and adheres to the stigma (*inset*, *red arrow*). In both cases, pollen moves against gravity force

During the bee’s final approach to the flower, electrostatic forces between these two bodies grow rapidly larger (Fig. 5). This has been suggested to have important consequences for pollen transfer from anther to bee and back from bee to stigma (Corbet et al. 1982; Erickson and Buchmann 1983; Vaknin et al. 2000, 2001). In addition, indeed, electrostatic pollen transfer can be demonstrated experimentally by bringing a charged acrylic rod in close vicinity to a pollen-bearing stigma (Fig. 6). In this experiment, bidirectional transfer from anther to charged rod and charged rod to stigma is clearly apparent, and depends upon the polarity of applied charge.

**Fig. 7:**
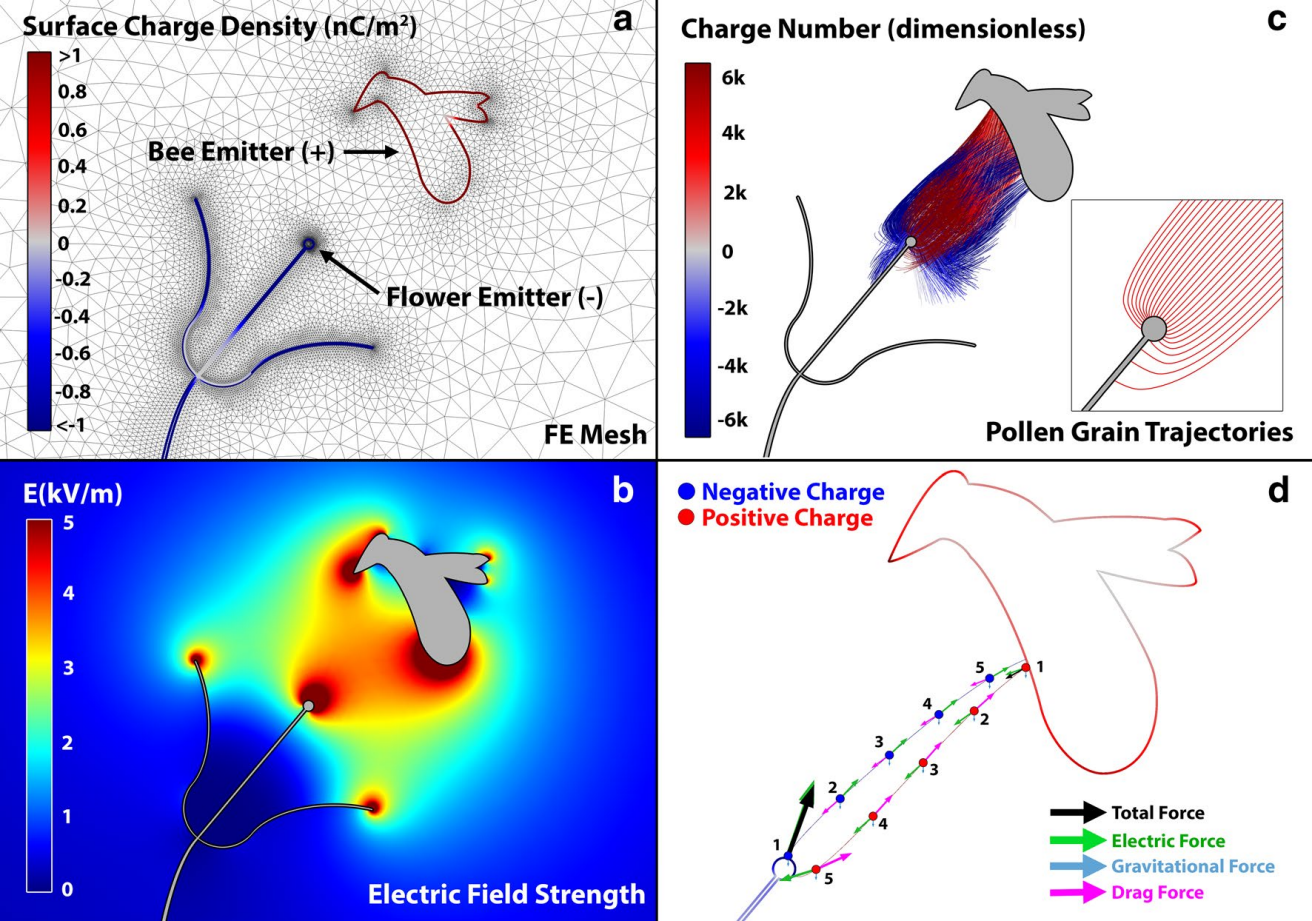
Finite-element modelling and visualisation of bee/flower electrostatic interaction in pollen transfer. **a** Model construction, showing the finite-element optimised mesh, and surface charge density (*scale bar*, nC/m^2^). Bee is set up as a positively charged pollen emitter. The negatively charged flower emitter can be regarded as a stigma or an anther in this model. **b** Model solution, showing the electric field (kV/m) arising between bee and flower, resulting in electric field hot spots, where electrical forces on charges are higher. The APG (100 V/m) is included in the model. **c** Simulation of bidirectional pollen grain transfer between flower and bee, taking into account gravitational, electrical, and viscous drag forces. Trajectories of 2000 pollen grains between bee and stigma are shown (*blue* and *red lines*). Pollen travelling from the bee to the stigma is positively charged while pollen originating on the flower and travelling to the bee negatively charged. Pollen charges are uniformly distributed between −40 and +40 fC. *Lines* show the pollen trajectories and the colour of the line shows the charge number of the pollen (a dimensionless quantity equal to the total charge divided by the charge of a proton). *Inset* is a closer view of the paths of 25 positively charged pollen grains converging towards the stigma due to its high negative charge density. **d** Close-up finite-element visualisation of two pollen grains during their opposite journey from bee to flower (*blue*) and flower to bee (*red*). Pollen grain colour indicates its charge (*red* positive, *blue* negative). *Green arrows* show the electric force, cyan the gravitational force, and magenta the drag force. *Black arrows* show the total resultant force. The trajectory for each of the two pollen grains is shown at 50 ms intervals from *t* = 0 (position 1) to *t* = 200 ms (position 5). Force arrows are drawn to scale

Just as morphology, geometry and specific impedance of structures are key influences on the electric field at the scale of trees and plants (Fig. 2), this remains true at the smaller scale of a flower’s anther, stigma, and pollen grain. Simply due to their geometry, protruding floral structures such as a long stigma will create a strong local electric field (Fig. 7a, b), and indeed, flowers with a flat corolla and long stigma appear to capture more pollen through electrostatic forces than those with concave corolla and shorter stigma (Vaknin et al. 2001). However, the specific impedance and connectivity to electrical ground will also influence the electric field around different floral components. Measurements from oil seed rape flowers (*Brassica napus*) show the stigma and outer nectaries to have lower impedance paths to earth than the petals, sepals, styles, and anthers (Corbet et al. 1982), resulting in relatively greater charge density by induction at these points (Fig. 7a, b).

Knowing that electrostatic forces can influence pollen transfer (Fig. 6) and that morphology, conductivity, and overall geometry influence the forces generated, how can we best describe their impact on pollen transfer in the flower–bee interaction? The modelling approach, again, has yielded the greatest insights so far. Bowker and Crenshaw (2007a, b) analytically modelled the motion of pollen charged to between 0.1 and 40 fC as it falls under gravity and determine that electrostatic forces can cause plants to draw in and capture pollen that would otherwise be lost under the influence of gravity, from significant distances away (cf >20 mm for pollen carrying 10 fC of charge). We have here constructed another quantitative model, extending the flower–bee finite-element model outlined in the previous section (Fig. 5) to include pollen grains (Fig. 7).

In the model, we select one of many possible approach trajectories that could be taken by the bee to the flower and set a range of parameters for a sum of 2000 pollen grains. Pollen grains are initially stationary in the model and located either attached to the flower (where they have a negative charge of between 0 and −40 fC), or attached to the ventral side of the bee (where they have a positive charge of between 0 and +40 fC). All grains had a mass (m) between 1 and 5 ng. There were three forces included in the model that influence the motion of individual grains: gravitational forces (G*m in the downward direction), electric forces (using Coulomb’s law), and viscous drag (in low-Reynolds number fluid). Pollen acceleration is modelled as the sum of the forces on the grain divided by the grain’s mass.

The results of the model show pollen grains of masses up to and including the mass of maize pollen undergo significant electrical forces, with the strong electric field between flower and bee more influential than gravity (Fig. 7). Pollen readily moves against gravitational forces, both from plant to bee and from bee to plant under the influence of electrical forces. When we look in detail at the trajectory of just two pollen grains during the bee–flower interaction, it is possible to see the contributions of each of these three forces at successive moments in time (Fig. 7c). For pollen grain one, at position one (on the bee, *t* = 0 ms), there is a repulsive electrostatic force (green arrow) acting on the positive pollen grain in the direction normal to the surface of the bee (also positively charged). The grain reaches terminal velocity almost instantaneously as viscous drag forces in the opposite direction to the velocity of the grain, quickly balance the other forces. Fifty milliseconds later, the pollen grain has moved closer to the stigma due to electric forces and gravity. Here, at point 2, the drag force perfectly opposes these other two forces, and the grain moves at terminal velocity. At this point, the grain is following a trajectory that would take it below and past the stigma (points 3 and 4), but as it approaches the stigma, the floral electric field rapidly increases (Fig. 7b, red zones). This intensification in the electric field at point 5 (*t* = 200 ms) accelerates the grain towards the stigma where it finally makes contact and is no longer simulated. The second grain’s journey is similar but in the opposite direction. Here, the initial force from the plant is again repulsive (negative pollen and negative plant), followed by attractive forces from the opposite surface (bee). Gravity plays a minor role in determining the trajectory of the pollen grains and simulation with many (2000) grains with varied initial location, charge or mass shows that almost all, if free to move, will reach either the protruding sexual organs of the flower or the hairy surface of the bee (Fig. 7c), as predicted by Armbruster (2001), Corbet et al. (1982), and Vaknin et al. (2000, 2001).

This simulation highlights the non-random and organised directed trajectory of pollen grains under electrostatic forces. Considering the picture generated by both empirical and modelled data, some questions naturally arise. Are plants morphologically and physiologically adapted to increase or take advantage of these electrostatic effects? Do some flower species actively generate or control the structure and dynamics of the electric field surrounding them? Are floral electric fields adapted, much like colours and fragrances are, to signal resource provisioning to bees, or their readiness for pollination? Given that bees can perceive detail within the floral electric field (Clarke et al. 2013), we propose that such adaptation is plausible. Electrostatics and electroreception are likely to be a *bona fide* part of the multiple co-evolutionary adaptations between plants and their pollinators. For example, Armbruster (2001) suggests that there is an adaptive compromise at work, where increasing the length of the stigma also increases the risk of damage to the structure as it is less well protected by the outer structure of the flower. Thus, from an electrostatic perspective, there seems to be clear benefits to pollination for the flat corolla, tall stigma morphology, though only some flowering plant species have converged on this shape.

## Conclusions and future directions

As a recently defined field, there is still a vast amount that is unknown in the electrical ecology of pollination, each from the perspectives of the bee the flower and the electric field. Finite-element models are proving a powerful tool in describing electrical interactions between the bee and the flower, but we need empirical data to confirm their predictions under a broader range of conditions. In many cases, the influence of electric fields may be strongly reduced by environmental factors not simulated in our models. High humidity, for example, leads to the formation of moist films on surfaces which prevent charge build up, and a dense rainforest canopy acts like a Faraday cage, negating the influence of the atmospheric electric field at ground level, possibly preventing the formation of floral electric fields altogether. How do these different environmental conditions affect the foraging behaviour of pollinators? At the scale of the pollen grain, even small humidity gradients in the floral headspace (von Arx et al. 2012) may influence the strength of electric forces on pollen grains. Does this impact the efficacy of pollen transfer? Another mitigating factor in pollen transfer is high winds. It is estimated that wind forces overpower electrical forces at high wind speeds (>10 m/s) (Bowker and Crenshaw 2007b), but at lower speeds, electrical forces are a significant influence on pollen grain trajectories. Another major direction for future work should encompass how electrical information is used to inform animal behaviour. We know that pairing electrostatic cues with visual cues leads to faster learning in bumble bees (Clarke et al. 2013). Is this sensory modality an important part of the “sensory billboard” advertisement provided by a flower out in a field? Or could this cue be used to assess the profitability of individual flowers? Tarsal hydrocarbon “footprint odours” left by the previous visitors can indicate resource depletion (Saleh et al. 2007; Wilms and Eltz 2008), could their “electrostatic footprint” do the same?

The tripartite interactions between bees, flowers, and the electric field that exist all around them, reveal thus far underappreciated physical and sensory ecologies. These interactions are likely to be diverse and maybe ubiquitous among insects since all are subject to the laws that govern the force of electromagnetism. The electrical ecology of bees is not unique. Every flying pollinator of comparable size to a bee is subject to the same electrostatic forces from flowers and comparable forces from conspecifics and other species. Antennae and mechanosensory hairs are widespread in arthropods (Casas and Dangles 2010) as is the neural sensitivity required to detect minute forces on these structures (Yen et al. 1992; Shimozawa et al. 2003). The tendency to become positively charged is also not confined to bees. Insect cuticle in general displays this tendency (Edwards 1962). The generality of such processes remains to be explored in a wider range of species and sensory ecological contexts beyond pollination. The ubiquity of electric fields in the environment means that signals in this modality could potentially be used by a broad range of species in an array of contexts; from intraspecific communication to predator avoidance and prey detection.
